# Does the blunted stimulation of skeletal muscle protein synthesis by aging in response to mechanical load result from impaired ribosome biogenesis?

**DOI:** 10.3389/fragi.2023.1171850

**Published:** 2023-05-15

**Authors:** Thomas Chaillou, Diego Montiel-Rojas

**Affiliations:** School of Health Sciences, Örebro University, Örebro, Sweden

**Keywords:** anabolic resistance, resistance exercise, elderly, translational capacity, sarcopenia, muscle atrophy, rDNA transcription

## Abstract

Age-related loss of skeletal muscle mass leads to a reduction of strength. It is likely due to an inadequate stimulation of muscle protein synthesis (MPS) in response to anabolic stimuli, such as mechanical load. Ribosome biogenesis is a major determinant of translational capacity and is essential for the control of muscle mass. This mini-review aims to put forth the hypothesis that ribosome biogenesis is impaired by aging in response to mechanical load, which could contribute to the age-related anabolic resistance and progressive muscle atrophy. Recent animal studies indicate that aging impedes muscle hypertrophic response to mechanical overload. This is associated with an impaired transcription of ribosomal DNA (rDNA) by RNA polymerase I (Pol I), a limited increase in total RNA concentration, a blunted activation of AKT/mTOR pathway, and an increased phosphorylation of AMPK. In contrast, an age-mediated impairment of ribosome biogenesis is unlikely in response to electrical stimulations. In human, the hypertrophic response to resistance exercise training is diminished with age. This is accompanied by a deficit in long-term MPS and an absence of increased total RNA concentration. The results addressing the acute response to resistance exercise suggest an impaired Pol I-mediated rDNA transcription and attenuated activation/expression of several upstream regulators of ribosome biogenesis in muscles from aged individuals. Altogether, emerging evidence indicates that impaired ribosome biogenesis could partly explain age-related anabolic resistance to mechanical load, which may ultimately contribute to progressive muscle atrophy. Future research should develop more advanced molecular tools to provide in-depth analysis of muscle ribosome biogenesis.

## 1 Introduction

Skeletal muscle plays a vital role in movement, body temperature regulation and inter-organ crosstalk. Aging can lead to sarcopenia, which is characterized by decreased muscle mass, strength, and physical performance (Cruz-Jentoft et al., 2019). Sarcopenia is associated with increased risk for adverse health events, including falls, loss of independence and chronic disability (Batsis et al., 2014; Cruz-Jentoft and Sayer, 2019). Muscle atrophy directly relates to loss of muscle strength (Nilwik et al., 2013). Thus, understanding the mechanisms behind age-related muscle atrophy is crucial to develop early preventive measures and mitigate sarcopenia risk in advanced aging.

The etiology of age-related atrophy is multifactorial but could mainly result from an imbalance between muscle protein synthesis (MPS) and muscle protein breakdown (Wilkinson et al., 2018). Cumulative evidence indicates that basal rates of MPS are not compromised in old compared with young adults (Volpi et al., 2001; Cuthbertson et al., 2005; Katsanos et al., 2005; Mayhew et al., 2009). Instead, an inadequate stimulation of MPS in response to anabolic stimuli [called anabolic resistance (AR)], including mechanical load associated with resistance exercise (RE), dietary amino acids (AA), and anabolic hormonal milieu could underlie age-related catabolic perturbations in protein homeostasis, leading to a progressive decline in muscle mass (Wilkinson et al., 2018). Recent reviews have analyzed the mechanisms linking AR and age-related muscle atrophy (Wilkinson et al., 2018; Prokopidis et al., 2021; Zhao et al., 2021; Kanova and Kohout, 2022).

Novel insights indicate that ribosome biogenesis is a major determinant of translational capacity and is involved in the control of skeletal muscle mass (Chaillou et al., 2014; Figueiredo and McCarthy, 2019; von Walden, 2019). To date, the impact of age-related AR to mechanical load on translational capacity is under debate. After providing a brief overview of the regulation of ribosome biogenesis, this mini-review will put forth the hypothesis that ribosome biogenesis is impaired by aging in response to mechanical load, which could contribute to the age-related AR and progressive muscle atrophy.

## 2 Brief overview of the regulation of ribosome biogenesis

The human 80S ribosome, a 4.3-MDa ribonucleoprotein complex, is composed of one small 40S subunit (40S-SU) and one large 60S subunit (60S-SU). The 40S-SU contains 18S rRNA and 33 ribosomal proteins (RPs), while 60S-SU is composed of 5S, 5.8S, and 28S rRNAs, and 47 RPs (Khatter et al., 2015). During protein translation, 40S-SU decodes the mRNA by promoting the interaction between the mRNA codons and the transfer RNA (tRNA) anticodons (aminoacyl-tRNA), and each added amino acid is then incorporated into the newly translated polypeptide by 60S-SU. Ribosomes are crucial for protein translation, which is determined by both translational efficiency (i.e., protein synthesis rate per ribosome) and translational capacity (i.e., number of ribosomes per unit tissue or total number).

**FIGURE 1:**
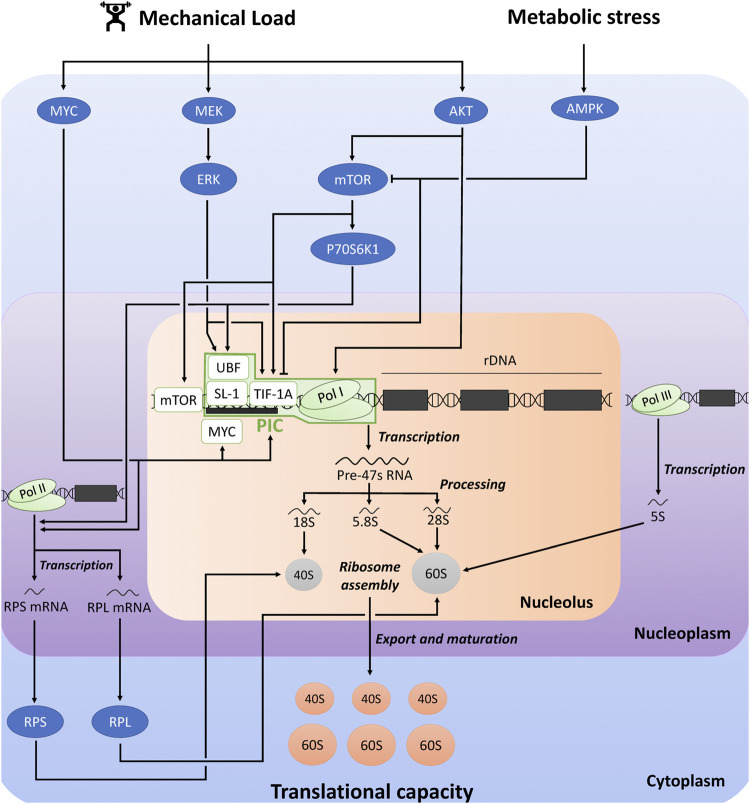
Schematic representation of the regulation of ribosome biogenesis and translational capacity in response to mechanical load and metabolic stress. The figure was adapted from previous reviews (Chaillou et al., 2014; Kusnadi et al., 2015; Figueiredo and McCarthy, 2019). AKT, protein kinase B; AMPK, AMP-activated protein kinase; ERK, extracellular signal-regulated kinase; mTOR, mechanistic target of rapamycin; PIC, pre-initiation complex; RPS, ribosomal protein small; RPL, ribosomal protein large; Pol, RNA polymerase; SL-1, selectively factor 1; TIF-1A, transcription initiation factor 1A; UBF, upstream binding factor.

Ribosome biogenesis is a multistep process leading to the *de novo* synthesis of ribosomes [for more detailed information, see (Thomson et al., 2013; Chaillou et al., 2014; Kusnadi et al., 2015; Figueiredo and McCarthy, 2019)]. It includes numerous steps (Figure 1): the transcription of ribosomal DNA (rDNA), mRNAs encoding RPs, and tRNAs, the translation of RPs, the processing of the polycistronic 47S rRNA precursor (47S pre-rRNA) into smaller rRNA (18S, 5.8S and 28S rRNAs), the assembly of rRNAs and RPs to form 40S- and 60S-SU, and the nuclear export and maturation of these subunits. Ribosome biogenesis requires the coordinated actions of all three RNA polymerases (Pol): RNA Pol I is responsible for the transcription of 47 pre-rRNA, RNA Pol II transcribes mRNAs encoding RPs (as well as all other mRNAs), and RNA Pol III transcribes 5S rRNA gene (as well as tRNA genes).

Pol I-mediated rDNA transcription is the rate-limiting step in ribosome biogenesis, and 47S pre-rRNA production is considered as the major regulatory point of ribosome biogenesis (Kusnadi et al., 2015). Pol I-mediated rDNA transcription is largely dependent on the expression and activity of the pre-initiation complex (PIC) at the rDNA promoter. PIC is composed of RNA Pol I complex, and the Pol I-specific transcription factors transcription initiation factor 1A (TIF-1A), selectively factor 1 (SL-1) complex [formed by the TATA-binding protein (TBP) and four TBP-associated factors (TAFs)], and upstream binding factor (UBF) (Gorski et al., 2007; Goodfellow and Zomerdijk, 2013). UBF first binds to the rDNA promoter and recruits SL-1 complex. UBF/SL-1 complex facilitates DNA binding and TIF-1A promotes the connection between these factors and Pol I.

Pol I-mediated rDNA transcription and the other steps of ribosome biogenesis are controlled by several signaling pathways which receive inputs from different cellular stimuli, including mechanical load, growth factors, hormonal, nutritional and metabolic influences [for more detailed information, see (Chaillou et al., 2014; Kusnadi et al., 2015; Brook et al., 2019; Figueiredo and McCarthy, 2019; Kirby, 2019; von Walden, 2019)]. The MAPK-ERK kinase (MEK)/extracellular signal-regulated kinase (ERK) and AKT/mechanistic target of rapamycin (mTOR) signaling pathways plays a critical role in regulating rDNA transcription. For instance, ERK and mTOR complex 1 (mTORC1) activates TIF-1A, and ERK and ribosomal protein S6 kinase 1 (P70S6K1) activates UBF (Kusnadi et al., 2015). Moreover, mTOR stimulates rDNA transcription through its association with the rDNA promoter (von Walden et al., 2016). AKT also promotes rDNA transcription by improving Pol I loading on rDNA promoter and transcription elongation, which are mediated by mTORC1-dependent and independent mechanisms (Chan et al., 2011). The transcription factor MYC is another key regulator of ribosome biogenesis, through its direct and indirect influences on Pol I-mediated rDNA transcription (Kusnadi et al., 2015). MYC stimulates the transcription of several factors associated with Pol I-mediated rDNA transcription, including the core factors of PIC (UBF, TIF-1A and Pol I subunits). Furthermore, MYC can translocate into the nucleolus where it binds to the rDNA promoter, thereby activating Pol I transcription. It can also interact with SL-1, which facilitates the stabilization of UBF/SL-1 complex and the initiation of rDNA transcription. Furthermore, AMP-dependent protein kinase (AMPK) is a crucial sensor of metabolic stress, which is activated in response to depletion of adenosine triphosphate (ATP) and accumulation of adenosine monophosphate (AMP). AMPK can directly and indirectly inactivate mTORC1 by phosphorylation (Hardie, 2008), which thus potentially impair rDNA transcription and ribosome biogenesis (Kusnadi et al., 2015). AMPK could also prevent Pol I-mediated rDNA transcription by inhibiting the interaction between TIF-1A and Pol I complex with the rDNA promoter, which blunts PIC assembly (Hoppe et al., 2009).

Although Pol I-mediated rDNA transcription is considered as the rate-limiting step of ribosome biogenesis, other regulatory steps could be influenced. RPs and other components of the translational machinery are translated from 5′-terminal tract of pyrimidine (5′-TOP) mRNAs. P70S6K was proposed to promote the translation of 5′-TOP mRNAs (Jefferies et al., 1997), while AMPK could suppress the translation of specific 5′-TOP mRNAs (Reiter et al., 2008). MYC could also promote the transcription of several genes encoding RPs of 40S-SU (ribosomal protein small, RPS) and 60S-SU (ribosomal protein large, RPL) (Coller et al., 2000; Kim et al., 2000).

## 3 Ribosome biogenesis in aged skeletal muscle in response to mechanical load

For the analysis of this mini-review, we selected studies comparing the level/expression of markers/regulators of skeletal muscle ribosome biogenesis between healthy young and healthy old humans/animals in response to mechanical load (RE in human studies; mechanical overload, and electrical stimulations in animal studies) (Table 1). We also included a few human studies combining RE and AA supplementation. These factors include markers of ribosome content/concentration (total RNA, rRNA, RPs), specific mediators of rDNA transcription by Pol I (TIF-1A, UBF, POL1s, TAFs, pre-47S rRNA) and important upstream regulators of ribosome biogenesis (MEK/ERK and AKT/mTOR pathways, MYC, AMPK) introduced in the previous section.

**TABLE 1:** Summary of the studies comparing the changes in ribosome biogenesis in response to mechanical load between young and old animals and subjects.

Animal studies				
References	Animals	Protocol	Main findings	Impaired ribosome biogenesis?
Chalé-Rush et al. (2009)	Y rats (8 M control, 8 M with surgery): 6 months	Mechanical overload induced by bilateral synergist ablation (28 days). PL and SO muscles	Lower increase in muscle mass (SO and PL) following 28 days of overload in O vs. Y rats. Similar increase in p-mTOR (Ser2448) levels in hypertrophied PLA muscle from both groups. p-mTOR (Ser2448) not affected by overload in SOL muscle. RPS6, p-P70S6K1 (Thr389) and p-ERK1/2 (Thr202) levels not affected by overload and aging in PLA muscle	Unclear
	O mice (8 M control, 8 M with surgery): 30 months			
Hwee and Bodine (2009)	Rats aged of 6, 9, 18, 26 and 30 months (6–8 M per age group; control animals and animals with surgery)	Mechanical overload induced by bilateral synergist ablation (7 and 14 days). PL muscle	Lower increase in muscle mass following 14 days of overload in rats aged >18 months vs. Y rats. Blunted increase in p-AKT (Ser473) levels of hypertrophied muscles in O (26 months) vs. Y (6 months) rats at 7 days of overload, with opposite result observed at 14 days	Potentially
Kirby et al. (2015)	Y mice (6 M per time point): 5 months	Mechanical overload induced by bilateral synergist ablation (up to 14 days). PL muscle.	Lower increase in muscle mass following 14 days of overload in O vs. Y mice. Diminished increase in 28S rRNA levels (3–7 days), pre-47S rRNA levels (3–7 days), and total RNA conc (µg/mg muscle) (5–14 days) in hypertrophied PLA muscle from O vs. Y mice. Higher levels of RP genes (*Rpl24*, *Rps19*, *Rpl10a*, *Rpl13* and *Rpl11*) in O vs. Y from 7 to 14 days of hypertrophy	Most likely
	O mice (6 M per time point): 25 months			
Thomson and Gordon (2005, 2006)	Y rats (7 M): 8 months	Mechanical overload induced by unilateral synergist ablation (7 days). PL and SO muscles.	Lower increased mass of PL muscle but not SO muscle at 7 days of overload in O vs. Y mice. Similar increase in p-AKT (Ser473), p-mTOR (Ser2448) and RPS6 levels in hypertrophied PL muscle of both groups, but lower increase in p-P70S6K (Thr389) levels in O vs. Y rats. Higher AMPK-α activity [p-AMPK-α (Thr172)/total AMPK-α] in hypertrophied PL muscle (but not in SO muscle) of O vs. Y rats	Potentially
	O rats (7 M): 30 months			
Haddad and Adams (2006)	Y rats (12 M): 6 months	Unilateral electrical stimulation inducing isometric contractions (3 contractions/min for 30 min). MG muscle	Lower absolute muscle mass in O vs. Y rats. Similar content of total RNA (µg/muscle) in non-exercised muscle in Y and O rats, with similar increase at 24–48 h post-electrical stimulation. Lower total conc (mg/g muscle) in non-exercised muscle in Y vs. O rats, which increased after electrical stimulation but remained lower in Y vs. old rats at 48 h. Lower levels of p-AKT (Thr308) in non-exercised muscle in O vs. Y rats, and increased p-AKT (Thr308) levels 24 h after electrical stimulation only in Y rats. Increase in p-P70S6K1 (Thr389) 24 h post-stimulation in both groups, but only in O rats at 48 h	Unclear
	O rats (12 M): 30 months			
Parkington et al. (2004)	Y rats (5 M per time point): 6 months	High-frequency electrical stimulation for 30 min. PL and TA muscles	Blunted increase in p-ERK1/2 (Thr202/Tyr204) in TA and PL muscles immediately after electrical stimulation (but not at 6 h) in O vs. Y rats. Low increase in p-mTOR (Ser2448) and in p-P70S6K (Thr389) levels (only in PL and TA, respectively) in O rats, but reported data not compared with Y rats	Potentially
	O rats (5 M per time point): 30 months			
West et al. (2018)	Y rats (6 M): 10 months	Acute unilateral electrical stimulation inducing eccentric contractions. TA muscle	Lower absolute muscle mass in O vs. Y rats. Similar increase in MPS up to 18 h post-stimulation in both groups, but lower at 48 h in O vs. Y rats. Similar increase in pre-47S rRNA (peak at 18 h) and total RNA conc (µg/mg muscle) (peak at 48 h) in both groups. Similar levels of p-P70S6K1 (Thr389), p-UBF (Ser637), Myc, Taf-1b in both groups	Unlikely
	O rats (6 M): 30 months			


Human studies				
References	Participants	Protocol	Main findings	Impaired ribosome biogenesis?
Brook et al. (2016)	Y physically active subjects (10 M): 23 years	*6-week unilateral RET, including 3 sessions/week: 6 x 8 KE at 75% 1-RM*. Acute RE at the beginning of RET: 6 × 8 KE at 75% 1-RM. VL muscle	Blunted hypertrophic response (i.e., impaired increase in thigh fat free mass and VL thickness) in O vs. Y subjects accompanied by a deficit in long-term MPS (6 weeks). Increase in total RNA conc (µg/mg muscle) only in Y males after 3 weeks of RET. Increase in p-P70S6K1 (Thr389), MYC, p-ERK1/2 (Thr202/Tyr204) and TIF-1A protein levels 75 min after the first bout of RE in Y males only, but no changes in total UBF and p-UBF (Ser484) levels, in p-mTORC1 (Ser2448), p-AKT (Ser473), and in p-TIF-1A (Ser649). No changes in mRNA levels for the 6 RP genes studied (except an increase in *RPS26* 75 min after the first bout of RE in O adults only), and in mRNA levels of *POL1RA*, *POL1RB*, *TAF1A*, *TIF1A* and *UBF*.	Most likely
	O physically active subjects (10 M): 69 years			
Farnfield et al. (2012)	Y physically active subjects (16 M): 20 years	*12-week RET, including 3 sessions/week: whole body, 50%–80% 1-RM*. Acute RE at the beginning and at the end of RET: 3 × 8 unilateral isokinetic KE at 60°/s. Exercise combined or not with 27 g of whey protein. VL muscle	Increase in p-P70S6K (Thr389) after RE (2 h) combined with protein ingestion more pronounced in O vs. Y subjects during the initial RE session, but not during the last session. Similar increase in p-mTOR (Ser2448) after RE (2 h) combined with protein ingestion in both groups during the initial session, while it was observed only in Y subjects during the last session. Similar levels of p-AKT (Ser473) in both groups after the initial and last RE sessions	Unclear
	O physically active subjects (15 M): 67 years			
Mayhew et al. (2009)	Y untrained subjects (21): 28 years	*16-week RET, including 3 sessions/week: 3 x 8–12 RM of squat, leg press and KE.* Acute RE at the beginning of RET: 3 x 8–12 RM squat, leg press and KE. VL muscle.	Similar increase in fiber CSA (type I and II) and thigh lean mass in both groups after RET. Increase in MPS (performed only on a subset of subjects) and p-AKT (Ser 473) levels compared to baseline at 24 h post-RE (first session) only significant in Y subjects, but no significant time × age interaction. Similar increase in p-P70S6K1 (Thr421/Ser424) in both groups at 24 h post-RE. pP70S6K1 (Thr389) not detectable	Unclear
	O untrained subjects (15): 64 years			
Drummond et al. (2008)	Y physically active subjects (7 M): 30 years	Acute RE: 8 × 10 KE at 70% 1-RM. 20 g EAA supplemented 1 h post-RE. VL muscle	Lower increase in MPS at 1–3 h post-RE in O vs. Y males, but similar increase at 3–6 h post-RE in both groups. Increase in p-AKT (Ser473) (3 h post-RE) and p-ERK1/2 (Thr202/Tyr204) (1 h post-RE) only in Y subjects. Higher levels of p-AMPKα (Thr172) in O vs. Y subjects at 1–3 h post-RE. Similar increase in p-mTOR (Ser2448) levels at 1–6 h post-RE in both groups. Trend to lower p-P70S6K1 (Thr389) levels at 1 h post-RE in O vs. Y subjects but overall, similar increase in p-P70S6K1 (Thr389) at 3–6 h post-RE.	Potentially
	O physically active subjects (6 M): 70 years			
Francaux et al. (2016)	Y physically active subjects (9 M): 22 years	Acute RE: 10 × 10 unilateral KE at 70% 3-RM. 30 g of whey protein ingested immediately after RE. VL muscle	Similar levels of p-mTOR (Ser2448) and p-AMPK (Thr172) after RE (30 min) in both groups. Trend to lower p-P70S6K (T389) levels in O vs. Y subjects after RE (30 min). Lower increase in p-AKT (Ser473 and Thr308) in O vs. Y subjects after RE (30 min)	Potentially
	O physically active subjects (6 M, 4 F): 69 years			
Fry et al. (2011)	Y physically active subjects (8 M, 8 F): 27 years	Acute RE: 8 × 10 KE at 70% 1-RM. 20 g EAA supplemented 1 h post-RE. VL muscle	Blunted increase in p-AKT (Ser473) at 24 h, p-mTOR (Ser2448) at 24 h, p-P70S6K1 (Thr389) at 6–24 h and MPS at 3–24 h in O vs. Y subjects after RE. Increase in p-ERK1/2 (Thr202/Tyr204) levels at 3–6 h post-RE only in Y subjects. No differences in RPS6 levels	Potentially
	O physically active subjects (8 M, 8 F): 70 years			
Kumar et al. (2009)	Y physically active subjects (25 M): 24 years	Acute RE: 3–6 sets unilateral KE and KF at 20%–90% 1-RM. VL muscle	Blunted increase in MPS and p-P70S6K1 (T389) levels in O vs. Y subjects after RE (1–2 h), but only at higher intensities (60%–90% 1-RM combined)	Potentially
	O physically active subjects (25 M): 70 years			
Lees et al. (2020)	Y healthy subjects (4 M, 3 F): 28 years	Acute RE: 8 × 10 unilateral KE at 70% 1-RM. 15 g EAA supplemented or not 20 min post-RE. VL muscle	Higher increase in p-AKT (Ser473) levels 2 h post-RE supplemented with EAA in O vs. Y subjects. No significant differences between groups for p-mTOR (Ser2481) and p-P70S6K1 (Thr421/Ser424) 2 h post-RE supplemented or not with EAA.	Unclear
	O healthy subjects (4 M, 3 F): 71 years			
Rivas et al. (2012)	Y healthy subjects (9 M): 27 years	Acute RE: 3 × 10 at 80% 1RM (LP and KE) VL muscle	Blunted increase in p-AKT (Ser473) and p-P70S6K1 (Thr389) in O vs. Y subjects at 0–6 h post-RE. No between-group differences for p-mTOR (Ser2448)	Potentially
	O healthy subjects (10 M): 74 years			
Stec et al. (2015)	Y physically active subjects (7M, 7F): 39 years	Acute RE: 9 × 10 KE at 65% 1-RM. VL muscle	Higher total RNA conc (ng/mg muscle) at baseline and 24 h post-RE in O vs. Y subjects. Tendency to higher levels of pre-45S rRNA at baseline in O vs. Y subjects, and significant increase in pre-45S rRNA 24 h post-RE only in Y subjects. Higher protein levels of RP (RPL3, RPL7a and RPS3, tendency for RPS6) at baseline in O vs. Y subjects, and tendency to increased levels at 24 h post-RE for RPS6 and RPS3 only in Y subjects. Higher levels of TIF-1A and p-P70S6K (Thr421/Ser424) at baseline in O vs. Y subjects, with increased TIF-1A levels 24 h post-RE only in Y subjects and decreased in p-P70S6K (Thr389) 24 h post-RE in O subjects. Similar increase in p-mTOR (Ser2448) 24 h post RE in both groups. No changes in UBF and MYC.	Most Likely
	O physically active subjects (6M, 6F): 76 years			

conc, concentration; CSA, cross-sectional area; EAA, essential amino acid; F, female; KE, knee extension; KF, knee flexion; LP, leg press; M, male; MG, medial gastrocnemius; MPS, muscle protein synthesis; O, old; p-, phosphorylated; PL, plantaris; RE, resistance exercise; RET, resistance exercise training; RM, repetition maximum; RP, ribosomal protein; SO, soleus; TA, tibialis anterior; VL, vastus lateralis; Y, young.

### 3.1 Animal studies

#### 3.1.1 Mechanical overload

Several rodent studies indicate that aging impairs muscle hypertrophic response to mechanical overload induced by synergist ablation (Thomson and Gordon, 2005; Thomson and Gordon, 2006; Chalé-Rush et al., 2009; Hwee and Bodine, 2009; Kirby et al., 2015). This was accompanied by a lower increase in total RNA concentration in hypertrophied plantaris muscle from old compared with young mice (Kirby et al., 2015). This difference in total RNA concentration (µg/mg muscle) implies that total RNA content (µg/muscle) was reduced in old vs. young hypertrophied muscles due to the differences in muscle mass between the two groups. Given that rRNAs constitutes the largest proportion of total cellular RNA (ranging from 70% to 90%), reduced total RNA content indicates a decreased translational capacity mediated by impaired ribosome biogenesis (Chaillou et al., 2014; Figueiredo and McCarthy, 2019). Kirby et al., found that pre-47S rRNA and 28S rRNA levels paralleled the changes in total RNA concentration. In addition, they observed that the expression of numerous RP genes similarly increased in young and old hypertrophied muscles until 5 days of overload, after which some of these RP genes (especially Rpl11) remained only highly expressed in old muscles subjected to mechanical overload. This finding is consistent with previous results showing that the expression of numerous RP genes was negatively correlated with the increase in human lean mass after 20 weeks of resistance exercise training (RET) (Phillips et al., 2013). Although it remains to be investigated, impaired induction of ribosome biogenesis with age in response to mechanical load might be associated with changes in RP translation (all or specific RPs).

The activation of AKT/mTOR pathway is also attenuated by aging during the first week of overload (Thomson and Gordon, 2006; Hwee and Bodine, 2009). The increase in AKT phosphorylation in hypertrophied plantaris muscles (7 days of overload) was impaired in old compared with young rats (Hwee and Bodine, 2009), a result not confirmed in another study (Thomson and Gordon, 2006). However, the latter study observed that overload-mediated increase in P70S6K phosphorylation was attenuated in plantaris muscles from old vs. young rats. In addition, AMPK-α phosphorylation status (phosphorylated/total forms) was more elevated in overloaded plantaris muscles (but not in overloaded soleus muscles) from old than young rats, which is consistent with the diminished overload-induced hypertrophy by aging only observed in plantaris muscles (Thomson and Gordon, 2005). Furthermore, the attenuation of muscle hypertrophy by aging following prolonged overload (28 days), was not associated with age-differences in translational signaling (mTOR pathway and ERK1/2 phosphorylation) (Chalé-Rush et al., 2009). Altogether, aging blunts muscle hypertrophy induced by synergist ablation, and this corroborates with an altered ribosome biogenesis, as evidenced by a lower ribosome content (i.e., rRNA content), and an impaired rDNA transcription and blunted anabolic pathways during the early phase of muscle growth.

#### 3.1.2 Electrical stimulation

To our knowledge, no studies have investigated in a more physiological model inducing mild muscle growth, whether age-related attenuation of muscle hypertrophy is directly associated with impaired ribosome biogenesis. Some studies assessed markers/regulators of ribosome biogenesis following a single bout of electrical stimulations (Parkington et al., 2004; Haddad and Adams, 2006; West et al., 2018), a physiological model of RE known to stimulate MPS (Wong and Booth, 1990; West et al., 2018) and to moderately increase muscle mass (Baar and Esser, 1999). Following maximal eccentric contractions induced by electrical stimulations, MPS similarly increased in young and old rats at 18 h, while it remained only elevated in young animals at 48 h (West et al., 2018). No age-related differences were found for pre-47S rRNA, total RNA concentration, P70S6K1 and UBF phosphorylations, and Myc and Taf-1b mRNA. These findings suggest that ribosome biogenesis is not impaired by aging in response to electrical stimulation, and this cannot explain the age-mediated attenuation of MPS observed at 48 h. In another study using maximal isometric contractions induced by electrical stimulation (Haddad and Adams, 2006), total RNA content similarly increased at 24–48 h following exercise in young and old rats. Furthermore, the activation of several upstream regulators of ribosome biogenesis was impaired by aging (AKT, ERK1/2, mTOR, P70S6K) in response to electrical stimulation (Parkington et al., 2004; Haddad and Adams, 2006). Despite these results, the absence of specific impact of aging on ribosome content and Pol I-mediated rDNA transcription suggests that ribosome biogenesis is not altered by aging after a single bout of electrical stimulation. Further studies should investigate the effect of aging on muscle hypertrophy and translational capacity in response to chronic electrical stimulation.

### 3.2 Human studies

#### 3.2.1 Training intervention

Currently, only one study investigated the causes of age-related AR and attenuated muscle hypertrophic growth during a training intervention with a focus on ribosome biogenesis (Brook et al., 2016). Following 6 weeks of unilateral RET, muscle hypertrophy was blunted in old compared with young men, and this was accompanied by a deficit in long-term MPS. An increase in total RNA concentration was found in young but not in old males following 3 weeks of the training intervention, and similar results were observed for phosphorylated P70S6K1 and ERK1/2, MYC, and TIF-1A 75 min after the first RE session. Noteworthy, other factors associated with ribosome biogenesis were unaffected by aging in this study (phosphorylated forms of Akt, mTORC1, UBF, and TIF-1A, and 5 RP genes), while *RPS26* mRNA only increased in old males 75 min after the first RE session (Table 1). Although blunted muscle hypertrophy and AR mediated by aging are certainly multifactorial, impaired ribosome biogenesis (and more likely rDNA transcription) seems to be one contributing factor of this age-related deficit. In another study, no clear age-differences in MPS and translational signaling (AKT and P70S6K1) were found 24 h following the first session of a 16-week RET program, which is consistent with the similar gains in lean mass and fiber size observed in young and old subjects after RET (Mayhew et al., 2009). Although type-II muscle fibers were smaller in old than in young individuals in this study, the absence of age-related differences in hypertrophic response is surprising and is in contrast to most studies published in the field (Welle et al., 1996; Kosek et al., 2006; Greig et al., 2011; Peterson et al., 2011; Mero et al., 2013; Brook et al., 2016). An age-related effect on translational signaling associated with ribosome biogenesis (AKT, mTOR and P70S6K) was also uncertain in another study where muscle biopsies were collected after the first and last sessions of a 12-week RET (Farnfield et al., 2012). Unfortunately, clear conclusions cannot be drawn from this study because changes in muscle size after RET were not assessed.

Recently, the magnitude of RET-induced muscle hypertrophy was assessed in relation to changes in ribosome biogenesis in old subjects (65 years of age) (Stec et al., 2016). Unfortunately, this study did not include a control group of young participants (study not presented in Table 1 for this reason). Following 4 weeks of RET (3 sessions/week), total RNA concentration increased in moderate and extreme responders, but not in nonresponders. RET led to a more robust increase in MYC levels in moderate and extreme responders compared with nonresponders, and rRNA abundance only increased in extreme responders after RET. Other studies have proposed that the extent of ribosome biogenesis may be predictive of the hypertrophic response irrespective of age (Kim et al., 2007; Figueiredo et al., 2015), while blunting Pol I-mediated rRNA transcription abolishes *in vitro* hypertrophy in human cells (Stec et al., 2016). Altogether, these findings support the idea that impaired ribosome biogenesis (especially Pol I rDNA transcription) may contribute to the limited hypertrophic response observed in aged populations displaying a chronic deficit in MPS during RET.

#### 3.2.2 Single resistance exercise session

Currently, only one study compared Pol I-mediated rRNA transcription between old and young subjects following a single RE bout (Stec et al., 2015). Increased pre-45S rRNA levels 24 h following RE were observed in muscle from young but not old individuals. This was associated with a concomitant elevation of TIF-1A protein in the young group only, while UBF and MYC levels remained unchanged. Intriguingly, muscles from older participants had at baseline higher total RNA concentration, levels of several RPs and TIF-1A, and tended to present greater levels of pre-45S rRNA in comparison with younger participants. These results are in agreement with the higher muscle RNA concentration found in old than in young rats (Haddad and Adams, 2006) (Table 1), which according to Stec et al. (2015), might indicate an age-related accumulation of ribosome (potentially dysfunctional) at rest. Another possibility is that ribosome content (number/muscle) is not affected by aging in rested muscle, but its concentration (number/unit muscle) increases due to muscle atrophy (Figueiredo and McCarthy, 2019). This idea seems plausible since RNA content was similar in young and aged muscles at baseline, at least in rodents (Haddad and Adams, 2006). These two discordant hypotheses underline that accurate normalization of biological data in conditions of marked changes of muscle mass is crucial to properly interpret scientific results and avoid misleading conclusions (Chaillou et al., 2011).

Finally, other studies investigated the impact of aging on upstream regulators of muscle ribosome biogenesis after one RE session. An attenuated stimulation of MPS by aging is commonly observed following RE (Drummond et al., 2008; Kumar et al., 2009; Fry et al., 2011). This corroborates with an impaired activation of AKT/mTOR pathway and ERK1/2 (Drummond et al., 2008; Kumar et al., 2009; Fry et al., 2011; Rivas et al., 2012; Stec et al., 2015; Francaux et al., 2016), although one study did not confirm this result (Lees et al., 2020). AMPK phosphorylation was also higher in muscles from old compared with young participants at 1 and 3 h following RE (Drummond et al., 2008), which was not confirmed in another study ∼30 min after RE (Francaux et al., 2016). Factors such as protein supplementation (only applied in the latter study) and the time-points of tissue collection might explain the discrepancy between the two studies. In summary, the human studies focusing on the acute response to RE suggest that aging impairs Pol I rDNA transcription and limits the activation/expression of several upstream regulators of ribosome biogenesis, which may contribute to the attenuated stimulation of MPS.

## 4 Conclusion, perspectives and future research directions

Aging appears to impair muscle hypertrophic response to mechanical load in both animals and humans. This is associated with a deficit in long-term MPS (only studied in humans) and a blunted increase in RNA concentration. An attenuated acute stimulation of MPS and anabolic signaling by aging in response to mechanical load is commonly observed in animal and human studies. Although multifactorial, several lines of evidence indicate that AR to mechanical load partly results from the impaired induction of ribosome biogenesis (especially rDNA transcription by Pol I), which may contribute to the progressive decline in muscle mass with age.

The analysis presented in this mini-review focused on healthy aged humans and animals (Table 1). Aging is also associated with higher risks of developing diseases, such as cancer (White et al., 2014). Patients with advanced stage cancer are susceptible of suffering from cachexia, a wasting and multifactorial syndrome characterized by a severe loss of skeletal muscle mass with or without a reduction of body fat (Dodson et al., 2011). Skeletal muscle wasting is accompanied by a blunted protein synthetic capacity in preclinical models of cancer cachexia (Brown et al., 2018; Kim et al., 2021). This muscle anabolic deficit corroborates with an impaired ribosomal production in several models of cancer cachexia, including ovarian cancer (Kim et al., 2021), colorectal cancer (Kim et al., 2022), and lung cancer (Belcher et al., 2022). Specifically, a reduced rRNA concentration, and an impaired Pol I-mediated rDNA transcription initiation and/or elongation were found in these three types of cancer (Kim et al., 2021; Belcher et al., 2022; Kim et al., 2022). Nevertheless, some divergences in ribosomal capacity were observed in these studies, which were likely due to differences in tumor types (e.g., LP07 and LLC models of lung cancer) and tumor burdens (e.g., metastatic and xenograft tumors) (Belcher et al., 2022; Kim et al., 2022). Although these findings remain to be confirmed in clinical studies with cancer patients, impaired ribosome biogenesis could contribute to anabolic deficits associated with cancer cachexia. Chemotherapeutic agents, which are commonly used to treat cancer and blunt tumor progression, have also a detrimental effect on skeletal muscle mass (Barreto et al., 2016; Damrauer et al., 2018). A recent study showed in C2C12 myotubes that exposure to chemotherapeutic agents reduced protein synthesis, rRNA content, and Pol I-mediated rDNA transcription at the initiation step, suggesting that chemotherapy-induced anabolic deficits may partly result from impaired ribosome production (Guo et al., 2021).

Muscle disuse, which is commonly observed in older individuals due to frequent hospitalizations, immobilization, or bed rest, leads to a rapid loss of skeletal muscle mass and force (Urso et al., 2006; Kortebein et al., 2007). A recent study indicates in middle-aged rats (10 months) that muscle disuse (hindlimb suspension) reduces Pol I-mediated rDNA transcription, total RNA concentration and RNA synthesis, and increases RNA degradation (Figueiredo et al., 2021a). A reduced total RNA concentration was also observed in the latter study in healthy middle-aged men (50 years) after 2 weeks of knee immobilization. These findings provide strong evidence that translational capacity is impaired during muscle disuse, which could contribute to muscle atrophy. Since elderly population has higher risks of developing cancer and being hospitalized or immobilized, impaired ribosome biogenesis and translational capacity could be common mechanisms contributing to muscle wasting in aging population exhibiting sarcopenia, cachexia and muscle disuse.

The selected studies of our analysis essentially used standard methods (i.e., immunoblotting, quantitative Polymerase Chain Reaction, and spectrophotometry) to compare the level/expression of markers/regulators of ribosome biogenesis between young and old skeletal muscles in response to mechanical load. These methods are unfortunately limited to capture complex molecular events and provide an in-depth analysis of ribosome biogenesis. In addition, current knowledge on muscle ribosome biogenesis is recent (10–15 years) and has mainly focused on rDNA transcription. Future research should develop more advanced molecular tools and innovative technologies. For instance, proteomic analyses and translatomic methods such as ribosomal profiling could be used to assess changes in RP translation (Brar and Weissman, 2015). A recent study performed in skeletal muscle of older individuals (72 years) and combining ribosomal profiling and RNA-sequencing revealed a reduced translation of most mRNAs encoding for RPs in response to physical inactivity, while the opposite result was found after leucine ingestion (Mahmassani et al., 2021). Using identical methods, another study from the same group found in adult mice that leucine administration increased the translation of ribosomal transcripts (32 *Rpl* and 25 *Rps*) and the abundance of *Snord22* and *Snord82*, two small nucleolar RNAs essential for rRNA maturation and ribosome biogenesis (Drummond et al., 2017). Future studies should use these innovative tools to investigate whether RP translation and other regulatory processes of ribosome biogenesis are impaired with age in response to mechanical load. Because muscle cells do not exclusively contribute to global gene expression in a baseline state and during skeletal muscle hypertrophy (Murach et al., 2022), combining ribosome profiling with a ribosome-tagging approach in animal models would be of interest to answer the latter research question specifically in muscle fibers during aging (Doroudgar et al., 2019).

The results from Mahmassani et al. (2021) indicate that changes in RP translation observed in skeletal muscle from aged adults in response to physical inactivity or leucine ingestion are not specific to the small or large ribosome subunit. Nevertheless, a recent study performed in *Drosophila* showed that the mRNA levels of *RpS28a* and *RpS28-like* declined in skeletal muscle during aging (Jiao et al., 2021). In addition, the results reported in Table 1 suggest that aging increases the level of some specific RP transcripts in response to mechanical load in both animals (Kirby et al., 2015) and humans (Brook et al., 2016), and increases the abundance of some ribosomal proteins at baseline in humans (Stec et al., 2015). Since manipulation of specific ribosome proteins appears to influence skeletal muscle growth, at least *in vitro* (Chaillou et al., 2015), the possibility of an age effect on muscle ribosome composition, in particular in response to anabolic stimuli such as mechanical load, deserves further consideration. The concepts of ribosome heterogeneity and functional ribosome specialization are relatively recent and currently poorly explored in skeletal muscle (Chaillou, 2019). The impact of aging on muscle RP composition and stoichiometry of both polysomes and free subunits could be investigated by employing selected reaction monitoring-based proteomics and mass spectrometry using tandem mass tag technology (Shi et al., 2017). Finally, some other emerging fields such as genetic and epigenetic regulation of ribosome biogenesis (Figueiredo et al., 2021b) and ribophagy (Figueiredo et al., 2021a) would also be of interest to explore ribosome turnover in aged muscle.
